# Targeting WEE1 kinase as a p53-independent therapeutic strategy in high-risk and relapsed acute lymphoblastic leukemia

**DOI:** 10.1186/s12935-023-03057-8

**Published:** 2023-09-15

**Authors:** Hayden L. Bell, Helen J. Blair, Mankaran Singh, Anthony V. Moorman, Olaf Heidenreich, Frederik W. van Delft, John Lunec, Julie A. E. Irving

**Affiliations:** 1 Wolfson Childhood Cancer Research Centre, Translation and Clinical Research Institute, Newcastle University Centre for Cancer,, Newcastle Upon Tyne, UK; 2 Princess Máxima Center for Pediatric Oncology, Utrecht, The Netherlands; 3 Bioscience Institute, Newcastle University Centre for Cancer, Newcastle Upon Tyne, UK

**Keywords:** WEE1, Inhibitors, Leukemia, High-risk, Relapse, Translational, Cell cycle checkpoints

## Abstract

**Background:**

Outcomes for patients with relapsed acute lymphoblastic leukemia (ALL) are poor and there is a need for novel therapies to improve outcomes. Targeted inhibition of WEE1 with small-molecule inhibitor adavosertib (AZD1775) has emerged as a therapeutic strategy to sensitize cancer cells to DNA-damaging chemotherapeutics, particularly in the context of *TP53*-mutated tumors. However, WEE1 inhibition as a potential therapeutic strategy for patients with high-risk and relapsed ALL, including those with *TP53* mutations, has not been definitively evaluated.

**Methods:**

Anti-leukemic effects of adavosertib were investigated using a relapsed *TP53* isogenic cell model system, primary patient, and patient-derived ALL samples (*n* = 27) in an ex vivo co-culture model system with bone marrow-derived mesenchymal stem cells. Combination effects with drugs currently used for relapsed ALL were quantified by Excess over Bliss analyses. Investigations for alterations of cell cycle and apoptosis as well as related proteins were examined by flow cytometry and Western blot, respectively.

**Results:**

Our study demonstrates the potent anti-leukemic activity of the clinically advanced WEE1 inhibitor adavosertib in a large majority (*n* = 18/27) of high-risk and relapsed ALL specimens at lower than clinically attainable concentrations, independent of *TP53* mutation status. We show that treatment with adavosertib results in S-phase disruption even in the absence of DNA-damaging agents and that premature mitotic entry is not a prerequisite for its anti-leukemic effects. We further demonstrate that WEE1 inhibition additively and synergistically enhances the anti-leukemic effects of multiple conventional chemotherapeutics used in the relapsed ALL treatment setting. Particularly, we demonstrate the highly synergistic and cytotoxic combination of adavosertib with the nucleoside analog cytarabine and provide mechanistic insights into the combinational activity, showing preferential engagement of apoptotic cell death over cell cycle arrest. Our findings strongly support in vivo interrogation of adavosertib with cytarabine in xenograft models of relapsed and high-risk ALL.

**Conclusions:**

Together, our data emphasize the functional importance of WEE1 in relapsed ALL cells and show WEE1 as a promising p53-independent therapeutic target for the improved treatment of high-risk and relapsed ALL.

**Supplementary Information:**

The online version contains supplementary material available at 10.1186/s12935-023-03057-8.

## Background

Acute lymphoblastic leukemia (ALL) is an aggressive hematological cancer arising from the malignant transformation and aberrant self-renewal of B- and T-lineage lymphoid progenitors. Contemporary ALL therapy revolves around administration of multiple cytotoxic chemotherapeutic agents in several phases over several years, resulting in severe acute and long-term toxicities [1]. Although improvements to chemotherapy regimens and risk stratification have increased survival of patients with ALL, outcomes remain poor for patients that relapse and for elderly patients that cannot tolerate standard therapy [2–4].

*TP53* mutations represent a strong and independent predictor of treatment failure in ALL, with outcomes of patients harboring *TP53* mutant disease being particularly dismal [5, 6]. Although alterations of *TP53* are infrequent at disease presentation (< 15%) in ALL, they are significantly enriched at relapse (up to 30%) and in the high-risk low hypodiploid ALL subgroup (> 90%) [6–10]. Consequently, *TP53* mutated ALL remains a particularly challenging and, so far, unaddressed issue in the clinical management of ALL due to the limited therapeutic arsenal available.

Dysregulated p53 most often enables cells to circumvent the G_1_-S cell cycle checkpoint due to the loss of p53 target gene *CDKN1A* (encoding cyclin-dependent kinase (CDK) inhibitor p21) transcriptional induction by p53 in response to DNA damage. As a result, malignant cells become more heavily reliant upon the G_2_-M checkpoint to prevent inappropriate progression through mitosis with levels of DNA damage that would otherwise result in mitotic catastrophe and cell death [11]. As a critical gatekeeper of the G_2_-M checkpoint and key regulator of genomic integrity during S-phase, WEE1 represents a promising target for the development of anti-cancer therapeutic strategies. WEE1 functions as a dual-specificity kinase which selectively phosphorylates both Thr14 and Tyr15 (predominantly Tyr15) residues of both CDK1 and CDK2 to restrain their activation and halt cell cycle progression in the face of DNA damage. During DNA replication, WEE1 regulates appropriate initiation and progression of DNA replication forks and, thereby, prevents generation of deleterious DNA double-strand breaks [12]. As a means to tolerate replication stress (by chemotherapy, radiation, oncogenes) and limit excessive genomic instability, WEE1 is commonly over-expressed by malignant cells and its high expression has been associated with poor rates of survival in various cancer types [13–16]. Inhibition of WEE1 in combination with DNA-damaging agents has been explored as a strategy for tumors with dysregulated p53 and numerous pre-clinical and clinical studies demonstrate preferential sensitivity of *TP53* mutated tumors [17–19]; though this has been equally contested by other investigators in various different cancer types [20, 21]. However, few studies have investigated WEE1 inhibition as a therapeutic approach in ALL and, in particular, limited information is available regarding its potential in high-risk or relapsed ALL for which outcomes are poorest.

Here we demonstrate the effectiveness and molecular mechanisms of the potent and selective ATP-competitive small-molecule inhibitor of WEE1 adavosertib (also known as AZD1775 or MK-1775) in ALL, including ALL specimens bearing *TP53* and other prognostically poor genetic alterations. Further, we explore combination strategies of combining adavosertib with chemotherapeutics already in clinical use for ALL and provide evidence for the effective combination of adavosertib with the nucleoside analog cytarabine. Our results are of substantial therapeutic relevance, ascribing a critical role of WEE1 kinase activity in ALL blast survival, and support current development of adavosertib as a potential therapeutic strategy in high-risk and relapsed ALL.

## Materials and methods

### Cell culture

Jurkat, MOLT4, NALM6, RS4;11, and SEM cell lines were obtained from the American Type Culture Collection (Manassas, VA), authenticated by STR profiling, and regularly tested for mycoplasma contamination (cat. no. LT07-710, Lonza, Basel, Switzerland). The *TP53* isogenic NALM6 model was purchased from Horizon Discovery (Cambridge, UK). hTERT-immortalized mesenchymal stem cells (MSCs) were a gift from D. Campana (National University of Singapore, Singapore). Cells were cultured in RPMI-1640 (Sigma, St. Louis, MO) with 10% FBS and 2 mmol/L *L*-glutamine at 37 ℃ in humidified air supplemented with 5% CO_2_ and maintained in culture for no longer than 2 months and/or 25 passages.

### Clinical samples

Primary human ALL cells were purified by standard Ficoll-Hypaque density centrifugation from fresh and cryopreserved bone marrow aspirates of pediatric and adult patients (median age = 10.5 years) presenting or relapsing with ALL, and were confirmed by flow cytometry (Additional file 1: Table S2) to contain greater than 80% leukemic blasts. Samples were accessed through the Newcastle Haematology Biobank after appropriate informed consent in accordance with the Declaration of Helsinki (reference numbers 2002/111 and 07/H0906). PDX#10-r was kindly provided by O. Williams (UCL Great Ormond Street Hospital, London, UK). PDX#19-r was obtained from the Center for Patient Derived Models at Dana-Farber Cancer Institute (Boston, MA, sample id: DFAB-82241). Cytogenetic analyses of patient bone marrow aspirates were performed during routine clinical practice as previously described [22]. Patient clinical characteristics are provided in Additional file 1: Table S1. *TP53* mutation statuses of patient specimens were determined as described in Additional file Methods. Disease risk status was determined in accordance with National Cancer Institute guidelines.

### Animal studies

All regulated animal procedures were approved by the Animal Welfare and Ethical Review Board of Newcastle University (Newcastle upon Tyne, UK) and conducted in accordance with the Animals (Scientific Procedures) Act 1986 under the UK Home Office license *P74687DB5.*

### Patient-derived xenografts

Patient-derived xenografts (PDX) were generated as previously described [22]. Two to three mice per patient sample were transplanted with 5 × 10^5^ to 1 × 10^6^ viable ALL cells (Ficoll density gradient purified) by intrafemoral injection in 8–12 weeks old severely immunocompromised NOD.Cg-Prkdc^scid^Il2rg^tm1Wjl^/SzJ (NSG; RRID: BCBC_4142) male and female mice weighing 20–35 g. Mice were housed under specific pathogen-free barrier conditions with irradiated laboratory chow and sterile water ad libitum*.* Animals were exposed to a 12 h light/dark cycle. Leukemia progression was monitored by enumeration of human ALL blasts in the peripheral blood by flow cytometry using red cell lysis and antibodies detailed in Additional file 1: Table S2. PDX ALL blasts cells were isolated from bone marrow, spleen, or liver (PDX#11 only) of engrafted NSG mice.

### In vitro drug treatment and assessment of cytotoxicity in an hTERT-immortalized MSC co-culture model

All drugs were purchased from Adooq Biosciences (Irvine, CA) and dissolved in dimethyl sulfoxide (DMSO) vehicle.

For dose–response curves in cell lines, 8–10 × 10^3^ cells in 96-well format were treated with increasing concentrations of each compound for 96 h in technical triplicate. Relative proliferation was determined using resazurin-based alamarBlue reagent (ThermoFisher Scientific, Altrincham, UK). Dose response curves were fitted using non-linear regression to estimate IC50s.

Drug responses for primary and PDX ALL blasts were determined in an in vitro co-culture model using hTERT-immortalized primary bone marrow MSCs as previously described [23]. Briefly, primary ALL or PDX cells were co-cultured in flat-bottom 384-well plates (Greiner Bio-One, Stonehouse, UK) seeded with 2500 MSCs per well 16 h prior, with 1–3 × 10^4^ live ALL blasts per well in serum-free AIM-V medium (ThermoFisher Scientific). Cells were treated with indicated concentrations of drugs for 96 h in at least technical duplicate and live cells were enumerated by fluorescence image analysis with machine learning as described in ref. 23. Live cell numbers were normalized to respective vehicle-only controls.

Combination experiments were performed in a two-way matrix format using fixed ratios of drugs in an 8 × 8 matrix and 2- or threefold increasing drug concentrations. Drug combination effects were determined after 96 h, synergy scores were analyzed using the Bliss independence model [24, 25] and visualized using a Python script.

### Immunoblotting

For Western blot, cells were lysed in cold RIPA buffer (1% NP-40, 150 mmol/L NaCl, 5 mmol/L EDTA, 0.25% sodium deoxycholate, 50 mmol/L Tris–HCL pH 7.5, 0.1% SDS) supplemented with protease (Cat. No. 5892791001, Roche, Hertfordshire, UK) and phosphatase inhibitors (cat. no. 4906845001, Roche). Proteins were separated using 4–20% gradient SDS–polyacrylamide gels (Bio-Rad Laboratories, Hercules, CA), electrophoretically transferred to polyvinylidene difluoride membranes, and immunoblotted according to routine techniques [23]. Primary antibodies are summarized in Additional file 1: Table S2, all used with overnight incubation at 4 °C. HRP-conjugated secondary antibodies used for 1 h at room temperature were from Agilent (Stockport, UK) and included goat anti-mouse immunoglobulin-HRP and goat anti-rabbit immunoglobulin-HRP. Signals were visualized using enhanced chemiluminescence (GE Healthcare, Chicago, IL) on a Bio-Rad Gel Doc XR system. Densitometry was performed with ImageJ v2.1 (RRID: SCR_003070).

### Flow cytometric analysis

Apoptosis was analyzed using a fluorescein isothiocyanate (FITC)-propidium iodide (PI) Annexin V Apoptosis Kit (556547, BD Life Sciences, San Jose, CA) according to manufacturer’s instructions. The percentages of both early (Annexin V^+^/PI^−^) and late (Annexin V^+^/PI^+^) apoptotic cells were detected and measured using a FACSCanto II (BD Life Sciences) and analyzed using FlowJo^™^ software (BD Life Sciences; RRID:SCR_008520). Cell cycle analysis by PI (50 µg/mL) staining in the presence of RNase A (20 µg/mL; Sigma-Aldrich) was performed by flow cytometry using FlowJo software with gating to exclude cell debris and doublets. To assess premature mitotic entry, cells were fixed with 70% (v/v) ice-cold ethanol, permeabilized with 0.25% (v/v) Triton X-100 in phosphate-buffered saline, then stained with AlexaFluor 488-conjugated anti-phosho-histone H3 (pHH3, S10) antibody (Cell Signaling, Danvers, MA) at 4 °C in the dark. After PI staining for cell cycle analyses as above, the percentage and cell cycle distribution of pHH3-positive cells was determined by flow cytometry.

### Statistical analyses

Statistical analyses and graphing were performed with GraphPad Prism v9.5 (San Diego, CA, USA; RRID: SCR_002798) software or the Python programming language. Unless otherwise indicated, graphs represent the mean from a minimum of three biological replicate experiments and error bars portray the SEM. Two-tailed student *t*-tests were used to assess the statistical significance of differences in measurements between two groups. One-way ANOVA was used to compare three or more samples with a single variable. Two-way ANOVA was used to compare three or more samples with two variables. The post-hoc Tukey correction was applied to determine significance between any two conditions of multiple groups. Differences in AZD1775 IC50s between *TP53* wild type and mutant samples was calculated using the Mann–Whitney *U* test. Differences were considered statistically significant at *p < 0.05; **p < 0.01; ***p < 0.001; ****p < 0.0001.

Further details on the methods can be found in the Additional file information.

### Data availability

Data generated in this study are available upon request from the corresponding author.

## Results

### WEE1 kinase inhibition by adavosertib is cytotoxic as a single agent in ALL cell lines, independent of *TP53* mutation status

**Fig. 1:**
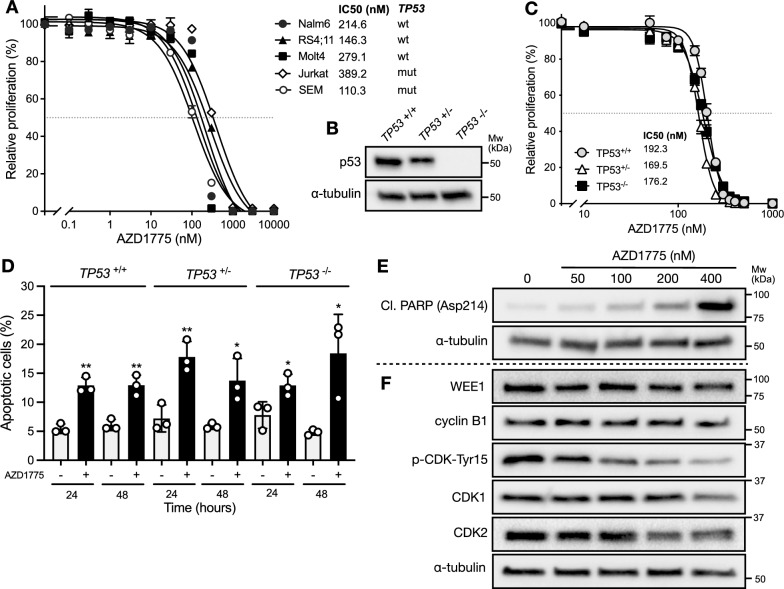
AZD1775 reduces cell viability and promotes apoptosis in a p53-independent manner in ALL cell lines. **A** Dose–response curves of ALL cell lines to AZD1775 for 96 h. Growth inhibitions were measured using a resazurin-based assay. Data were normalized to DMSO control and represent mean ± SEM of three independent experiments. **B** Immunoblot of p53 protein levels in the NALM6 *TP53* isogenic lines. **C** Dose–response curves of the NALM6 *TP53* isogenic lines to AZD1775 for 96 h. Data were normalized to DMSO control and represent mean ± SEM of three independent experiments. **D** Evaluation of apoptosis by Annexin-V staining in the NALM6 *TP53* isogenic lines treated with DMSO or 200 nM AZD1775 for 24–48 h. Error bars indicate mean ± SD of three independent experiments. **E** Immunoblot of apoptotic marker cleaved PARP (Asp214) in NALM6 cells treated for 24 h with DMSO or increasing doses of AZD1775. **F** Immunoblot of cell cycle markers in NALM6 cells treated for 6 h with DMSO or increasing doses of AZD1775. In **B**, **E**, and **F,** ɑ-tubulin was used as loading control and images are representative of three independent experiments

Firstly, we evaluated 5 established ALL cell lines with wild type (*n* = 3) or mutant (*n* = 2) *TP53* mutation status to determine sensitivity to the first-in-class, selective WEE1 kinase inhibitor adavosertib (AZD1775) (Fig. 1A). Each cell line was sensitive to adavosertib, as shown by 50% growth inhibitory (IC50) values, in a dose-dependent, sub-micromolar range (mean ± SD IC50 = 228 ± 111 nM). All cell lines showed complete cell growth inhibition by 1 µM; a concentration which is clinically achievable following oral administration of adavosertib [26, 27]. There was no apparent sensitivity difference between *TP53* wild type and mutant lines (*p* = 0.875).

To better investigate the role of p53 as a determinant of sensitivity to adavosertib, we used a *TP53* isogenic relapsed NALM6 B-ALL cell line model with wild type (*TP53*^+/+^), monoallelic knockout (*TP53*^±^), and biallelic knockout (*TP53*^−/−^) cells (Fig. 1B). Each isogenic line displayed comparable sensitivity to adavosertib, responding in a dose-dependent manner with no observable differences in IC50 values compared to the parental NALM6 line (mean ± SD IC50 = 179 ± 12 nM; Fig. 1C). Next we analyzed externalization of the apoptosis marker phosphatidylserine by flow cytometry to determine engagement of cell death in response to short-term drug exposure. In each isogenic line, adavosertib (200 nM) induced reproducible but modest (mean ± SD = 7.8 ± 2.7% increased) levels of apoptosis within 24 h as compared to vehicle-only treated cells and was not associated with a further time-dependent increase in apoptosis up to 48 h (Fig. 1D). Corroborating induction of apoptotic cell death was evidenced by concentration-dependent increased levels of the apoptotic marker cleaved PARP (Asp214) following 24 h exposure to adavosertib in each of the isogenic lines (Fig. 1E; Additional file 1: Fig. S1A).

Next we investigated whether sensitivity to adavosertib was associated with on-target killing by WEE1 kinase inhibition using CDK-Tyr15 phosphorylation as a pharmacodynamic response biomarker for WEE1 kinase activity [28, 29]. Exposure to adavosertib for 6 h consistently reduced phospho-CDK-Tyr15 levels relative to total CDK1 levels across each of the isogenic lines at IC50 concentrations (Additional file 1: Fig. S1B) and in a dose-dependent manner (Fig. 1F) while levels of cyclin B1 remained unchanged (*p* > 0.05).

These results suggest that on-target inhibition of WEE1 kinase activity by adavosertib can effectively induce apoptotic cell death in ALL cell lines as a single agent, independent of *TP53* mutation status.

### Adavosertib is cytotoxic in primary and primary-derived ALL blasts, independent of *TP53* mutation status

**Fig. 2:**
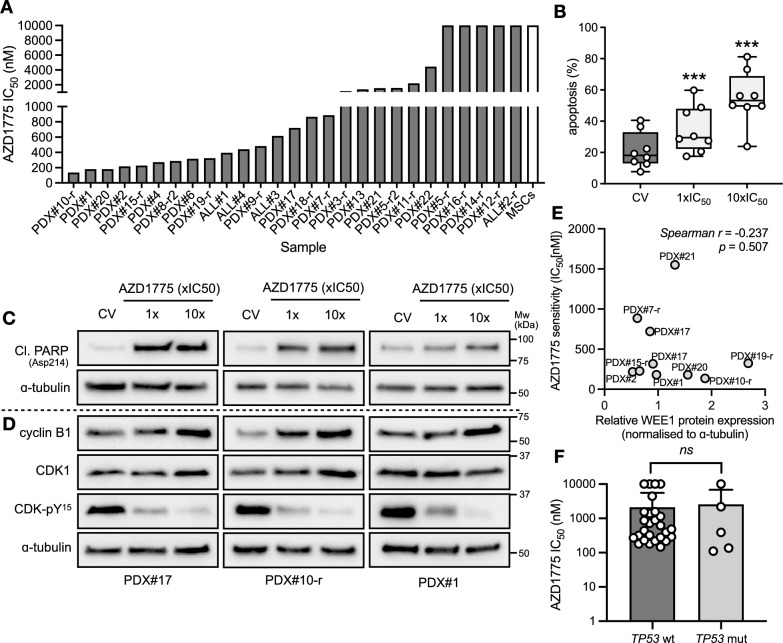
AZD1775 has antileukemic effects against primary and patient-derived ALL samples in vitro*,* independent of p53 status. **A** Sensitivity of primary (*N* = 4) and patient-derived xenograft (*N* = 23) ALL samples to AZD1775 exposure for 96 h in ex vivo co-culture with hTERT-immortalized MSCs. Drug responses were determined by fluorescence image-based microscopy in at least technical duplicate, relative to respective DMSO controls (*N* = *1* for each respective sample*)*. IC50 values are based on live cell enumeration fitted with a 4-parameter non-linear regression curve. MSC drug sensitivity is based on at least three independent experiments. **B** Evaluation of apoptosis by Annexin-V staining with flow cytometry in PDX samples (*N* = 8) following exposure to respective AZD1775 IC50 fractions for 48 h (*N* = 1 for each respective sample). Error bars show mean ± SD **C** Immunoblot of apoptotic marker cleaved PARP (Asp214) in PDX samples (*N* = 3) treated with respective AZD1775 IC50 fractions for 24 h. ɑ-tubulin was used as loading control. **D** Immunoblot of cell cycle markers in PDX samples (*N* = 3) treated with respective AZD1775 IC50 fractions for 24 h. ɑ-tubulin was used as loading control. **E** No correlation was observed between AZD1775 IC50 values and WEE1 protein expression levels determined by semi-quantitative immunoblotting (*n* = 10). The non-parametric one-tailed Spearman test was used to determine the correlation coefficient. **F** Statistical significance between adavosertib IC50s for cell lines, primary, and primary-derived ALL blast samples with (*N* = 27) or without (*N* = 5) *TP53* mutations were compared using Mann–Whitney U test. Dots in all panels represent individual samples

As cell lines often may not reliably reflect the characteristics of primary ALL, we next investigated the anti-leukemic potential of adavosertib in a panel of four primary and 23 patient-derived xenograft (PDX) specimens from patients including both relapsed and high-risk ALL subtypes (Additional file 1: Table S1). We utilized an ex vivo coculture model of ALL blasts on hTERT-immortalized mesenchymal stem cells (MSCs) to support short-term leukemic blast survival and growth, complemented by a fluorescence image-based microscopy platform to determine live cell numbers as previously described [23]. Adavosertib exposure resulted in a dose-dependent decrease in cell viability, with a mean ± SD IC50 of 0.86 ± 0.98 μM (Fig. 2A); though 5/27 (19%) samples did not attain 50% inhibition. Adavosertib IC50 values below approximately 1.4 μM, which is clinically achievable following oral administration of adavosertib [26, 27, 30], were observed in 18/27 (66%) of samples. This decreased viability was accompanied by significant induction of apoptotic markers phosphatidylserine (Fig. 2B) and cleaved PARP (Fig. 2C) following exposure to respective IC50 concentrations of adavosertib, highlighting the cytotoxic potential of single-agent exposure and indicating a consistent mechanism of cell death with the cell lines. By contrast, the bone marrow-derived MSCs were spared by adavosertib activity at concentrations effective in the leukemic blasts, despite evidence of on-target WEE1 inhibition (Additional file 1: Fig S2).

Consistent with the ALL cell lines, exposure to IC50 concentrations of adavosertib significantly reduced phospho-CDK-Tyr15 levels relative to total CDK1 levels in the PDX samples (*N* = *6, p* = 0.009), which was further diminished at higher adavosertib concentrations (*p* < 0.0001), confirming on-target WEE1 kinase inhibition in these cells (Fig. 2D). No significant differences were observed between treatment conditions for levels of total CDK1 or cyclin B1 (*p* > 0.05). Basal WEE1 protein expression, as measured by immunoblot and densitometry analysis, did not correlate with in vitro adavosertib sensitivity (Fig. 2E).

To better examine potential associations of adavosertib sensitivity with *TP53* mutation status, all investigated cell lines, primary, and PDX samples were compiled and stratified by *TP53* mutation status. *TP53* mutation statuses were confirmed by published literature for cell lines, and Sanger sequencing (Additional file 1: Table S3) and/or functional assessment by exposure to MDM2 inhibitor idasanutlin in primary and primary-derived samples (data not shown). Data indicated no significant difference between *TP53* mutation status and adavosertib sensitivity (*p* = 0.832; Fig. 2F).

These data indicate that WEE1 plays an important role in maintaining the survival of many ALL cells, independent of *TP53* mutation status, and may serve as a therapeutically targetable vulnerability in ALL.

### Adavosertib abrogates the G_2_-M checkpoint and induces S-phase cell cycle arrest in ALL blasts

**Fig. 3:**
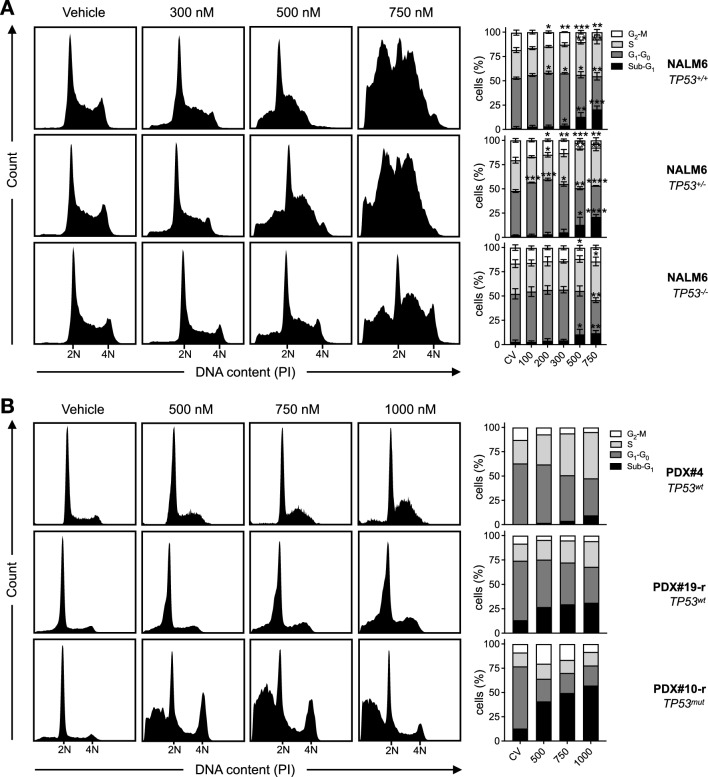
Pharmacological blockade of WEE1 by AZD1775 abrogates the G2-M cell cycle checkpoint and induces S-phase cell cycle arrest of ALL blasts. **A** Cell cycle analysis of a *TP53* isogenic NALM6 cell line model treated for 24 h with DMSO or increasing doses of AZD1775. Histograms are representative of three independent experiments. 2N DNA content indicates cells in G_0_ or G_1_ phase. 4N DNA content indicates cells in either G_2_ or M phase. Error bars show mean ± SD of at least three independent experiments*.* **B** Cell cycle analysis of high-risk *TCF3::HLF*-rearranged PDX#4 (*TP53*^*wt*^), relapsed B-other PDX#19-r (*TP53*^*wt*^), and relapsed hypodiploid PDX#10-r (*TP53*^*mut*^) treated with DMSO or increasing doses of AZD1775. Hypodiploid cell DNA content (< 2N) was normalized to diploid DNA content (2N). PDX#4, 24 h; PDX#19-r and PDX#10-r, 48 h. Data are representative of one independent experiment for each sample

Given the role of WEE1 in both the intra-S and G_2_-M cell cycle checkpoints, we determined how adavosertib might affect cell cycle kinetics using propidium iodide staining and flow cytometry. Using the *TP53* isogenic NALM6 model to assess the contribution of p53 mutation status, cells were exposed to 300–750 nM adavosertib for 24 h (Fig. 3A). Both p53-bearing *TP53*^+/+^ and *TP53*^±^ lines exhibited a dose-dependent decrease of cells with 4N DNA content indicative of a reduction in the number of cells in G_2_-M phase and an accumulation of cells with > 2N and < 4N DNA content, indicative of cell cycle arrest within S phase. Exposure to 750 nM adavosertib generated a severely abnormal cell cycle distribution with indistinct cell cycle phases, indicative of replicative catastrophe. By contrast, the p53-deficient *TP53*^−/−^ line did not exhibit a significant decrease in the proportion of cells with 4N DNA content at the same drug concentrations, though still exhibited substantial S-phase arrest by 750 nM. By analyzing the kinetics of histone H3 phosphorylation (pHH3), we deduced that NALM6 cells with < 4N DNA content were arrested in S-phase and were not cells which had undergone unscheduled mitosis without completing DNA synthesis and failed cytokinesis (mitotic catastrophe), as has been described in other cancer types in response to adavosertib (Additional file 1: Fig. S3A, B) [31]. Moreover, we did not observe increased DNA contents to over 4N, indicative of abnormal mitotic exits and increased numbers of multinucleate cells, a characteristic feature of mitotic catastrophe or endoreduplication [32]. While we observed increased expression of the replication stress marker γH2AX in response to adavosertib, this was completely diminished when cells were co-treated with the pan-caspase inhibitor zVAD.fmk suggesting elevated γH2AX levels at this time point may have occurred as a secondary marker of enhanced caspase-mediated nuclease activity in this model (Additional file 1: Fig. S4). Accordingly, each of the isogenic lines displayed significant sub-G1 DNA content upon flow cytometry analysis at clinically-attainable adavosertib concentrations (Fig. 3A), which is indicator of nuclear fragmentation and DNA degradation, further corroborating the induction of cell death.

To investigate the effects of adavosertib on ALL blasts of alternative genetic backgrounds, cell cycle analysis was also performed in high-risk *TCF3::HLF-*rearranged PDX#4 (*TP53wt*), relapsed B-other PDX#19-r derived from a 64-year old patient six-months post-bone marrow transplant (*TP53wt*), and relapsed low hypodiploid PDX#10-r (*TP53mut*) samples (Fig. 3B). Corroborating cell line data, adavosertib exposure induced a dose-dependent increase in the proportion of S-phase cells and corresponding decrease in G_2_-M phase cells by 24 h in PDX#4 and 48 h in PDX#19-r, with concomitant increases in sub-G1 DNA content. By contrast, PDX#10-r demonstrated increased G_2_-M phase cells and substantial accumulation of cells with sub-G1 DNA content by 48 h. While adavosertib exposure enhanced the proportion of pHH3-positive mitotic cells with 4N DNA content, we did not observe any changes to the proportion of pHH3-positive cells with less than or greater than 4N DNA content up to 48 h despite substantial cell death induction, further supporting the notion that ALL blasts do not undergo mitotic catastrophe in response to single agent adavosertib (Additional file 1: Fig. S3C).

Taken together, these data underscore a critical role of WEE1 kinase activity in both the intra-S-phase and G_2_-M cell cycle checkpoints of proliferating ALL cells which result in cell death via a mechanism not involving mitotic catastrophe.

### Adavosertib enhances the anti-leukemic activity of conventional chemotherapeutics used for the treatment of ALL

**Fig. 4:**
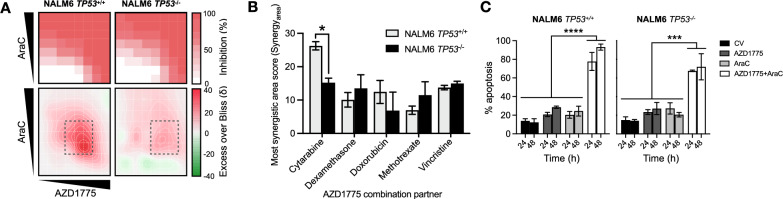
AZD1775 sensitizes ALL blasts to the nucleoside analog cytarabine and enhances apoptotic cell death. **A** Representative dose–response matrix analyses showing cell inhibition (top) and synergistic landscape (bottom) across diverse AZD1775-AraC dose combinations after 96 h in a NALM6 *TP53* isogenic model*.* Grey dashed boxes indicate the most synergistic area. **B** Pairwise combinations of AZD1775 with conventional ALL chemotherapy agents. Synergistic effects were quantified by most synergistic area scores as in **A**. Error bars indicate mean ± SD of three independent experiments in technical triplicate. **C** Evaluation of apoptotic by Annexin-V staining with flow cytometry in a NALM6 *TP53* isogenic model treated with AZD1775 (200 nM), AraC (15 nM), or their combination for 24 and 48 h. Error bars indicate mean ± SD of three independent experiments

Although adavosertib effectively induced apoptosis in ALL blasts as a single agent, it would most likely be incorporated into clinical trials in combination with chemotherapeutics used already in the relapsed or re-induction clinical ALL setting. As such, we investigated combination effects with five different classes of chemotherapeutics in the relapsed *TP53* isogenic NALM6 cell line model; these included the nucleoside analog cytarabine (AraC), the glucocorticoid dexamethasone, the anthracycline doxorubicin, the antimetabolite methotrexate, and the microtubule-targeting agent vincristine. To better model a clinical setting in which cancer cells are exposed to varied drug concentrations over time, we examined a wide range of drug dose combinations. Two-dimensional dose matrices were utilized for each pairwise drug combination and combination effects were determined using the Excess over Bliss synergy model [24, 25] at each combination dose ratio (Fig. 4A). Additive through to synergistic interactions were evident in each of the combination pairs (Additional file 1: Fig. S5), with the adavosertib-cytarabine combination attaining the highest synergistic interaction with most synergistic area scores (Synergy_area_) of 26.3 ± 2.2 and 15.2 ± 2.4 in the *TP53*^+*/*+^ and *TP53*^*−/−*^ lines respectively (Fig. 4B). The synergy observed with the adavosertib-cytarabine combination was significantly higher in *TP53*^+*/*+^ NALM6 cells (*p* = 0.015), though the p53-deficient *TP53*^−/−^ line still attained synergistic drug interaction suggesting p53 may be dispensable for the anti-leukemic effects of the combination. In support, the adavosertib-cytarabine combination induced significantly enhanced apoptosis as compared to either agent alone in both isogenic lines (*p* < 0.001; Fig. 4C). We also confirmed the robust synergistic interaction of adavosertib with clofarabine, an alternative nucleoside analog used in the relapsed ALL treatment setting (Additional file 1: Fig. S6A), achieving Synergy_area_ scores of 17.3 ± 7.8 and 11.2 ± 0.4 in the *TP53*^+*/*+^ and *TP53*^*−/−*^ lines respectively (Additional file 1: Fig. S6B).

### The adavosertib-cytarabine combination is active against primary-derived ALL blasts harboring various high-risk genetic aberrations

**Fig. 5:**
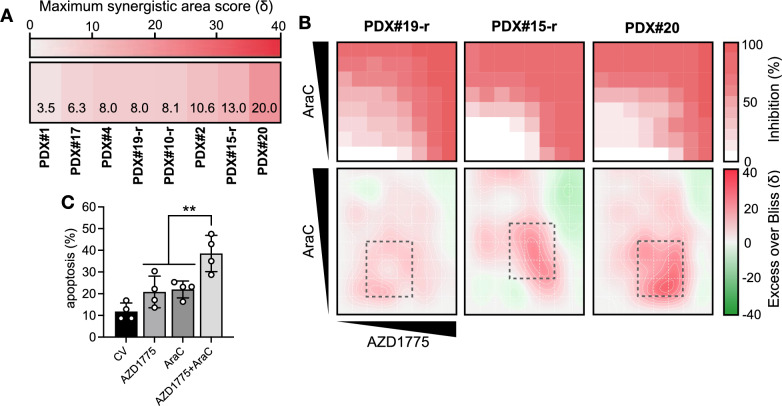
AZD1775 sensitizes primary-derived ALL blasts to cytarabine and enhances apoptotic cell death. **A** Drug combination interactions between AZD1775 and AraC were investigated in high-risk and relapsed PDX samples (*N* = 8) in in vitro co-culture with hTERT-immortalized MSCs for 96 h. Excess over Bliss synergy was determined at each dose combination and quantified by most synergistic area scores (*N* = 1 for each respective sample in at least technical duplicate). **B** Representative dose–response matrix analyses showing cell inhibition (top) and synergistic landscape (bottom) across a range of AZD1775-AraC dose combinations individualized for each PDX sample. Grey dashed boxes indicate the most synergistic area. **C** Evaluation of apoptosis by Annexin-V staining with flow cytometry in PDX#4, PDX#10-r, PDX#15-r, and PDX#19-r following exposure to respective IC50s of AZD1775, AraC, or their combination for 48 h. Dots represent individual samples. Error bars indicate mean ± SD of compiled PDX data (*N* = 1 for each respective sample)

Parallel combination studies were performed in PDX ALL, including relapsed and high-risk ALL subtypes (*KMT2A-*rearranged, iAMP21, low hypodiploid), in the co-culture MSC model. Overall, the adavosertib-cytarabine combination attained additive to synergistic anti-leukemic effects across the panel (mean Bliss Synergy_area_ = 9.7 ± 5.0, *n* = 8; Fig. 5A). The highest synergy was observed in presentation T-ALL sample PDX#20 (Bliss Synergy_area_ = 20.0). Analysis of the dose-interaction landscapes, however, indicated some regions of additive to antagonistic interaction in which combination activity did not surpass respective highest single agent responses (Fig. 5B).

Then we determined the capacity of the adavosertib-cytarabine combination to induce cell death in four representative relapsed ALL PDX samples. While exposure to either drug resulted in modest apoptosis (*p* < 0.018), combination treatment resulted in a greater than 2.3-fold increase in the proportion of apoptotic cells as compared to either single drug (*p* < 0.001) when normalized to the vehicle control (Fig. 5C). These results demonstrate that, at clinically achievable concentrations, the combination of adavosertib and cytarabine promotes apoptotic cell death rather than cell cycle arrest.

### WEE1 inhibition by adavosertib abrogates cytarabine-mediated cell cycle arrest and augments apoptotic cell death

**Fig. 6:**
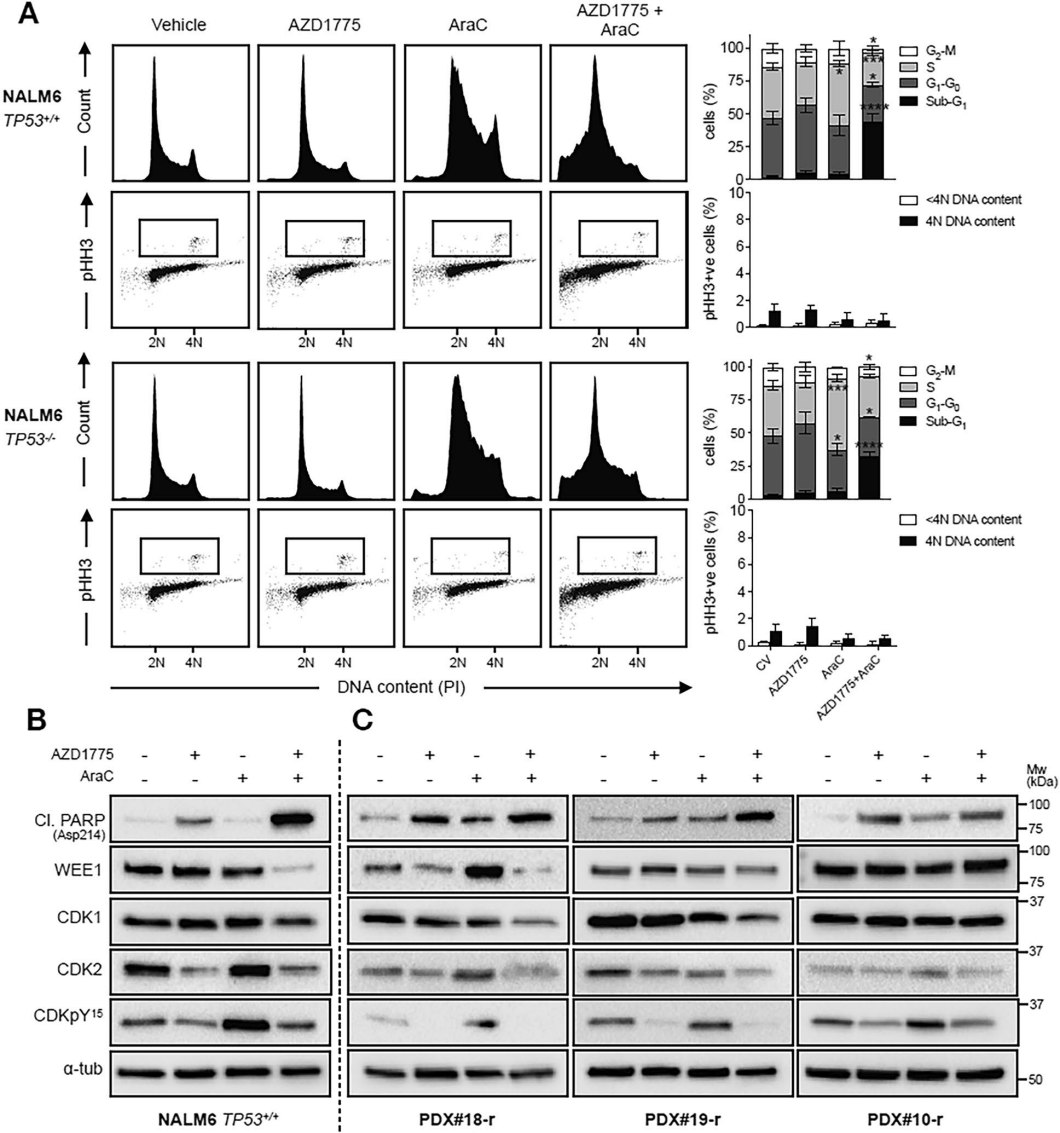
AZD1775 abrogates the intra-S-phase checkpoint and augments cell death induced by cytarabine. **A** A *TP53* isogenic NALM6 cell line model was treated for 24 h with AZD1775 (200 nM), AraC (15 nM), or their combination and subjected to dual cell cycle and pHH3 (mitotic cells) analysis using flow cytometry. Data are representative of three independent experiments. 2N DNA content indicates cells in G_0_ or G_1_ phase. 4N DNA content indicates cells in either G_2_ or M phase. Mitotic index was determined for cells with 4N and < 4N DNA content. Boxes indicate pHH3^+^ populations. Error bars show mean ± SD of at least three independent experiments*.* Top, PI alone; bottom, pHH3/PI. **B** Immunoblot of apoptotic and cell cycle markers in NALM6 cells treated for 24 h with AZD1775 (200 nM), AraC (15 nM), or their combination. Images are representative of three independent experiments. **C** Immunoblot of apoptotic and cell cycle markers in three relapsed PDX samples treated with respective IC50s of AZD1775, AraC, or their combination for 24 h. *N* = 1 for each respective sample. In **B** and **C**, ɑ-tubulin was used as loading control

Next we investigated the effects of the adavosertib-cytarabine combination on cell cycle progression in the NALM6 *TP53* isogenic cell line. While cytarabine significantly increased the S-phase sub-population in both isogenic lines, consistent with its mode of action as a nucleoside analog, this S-phase cell cycle arrest was abrogated upon co-treatment with adavosertib and their combination significantly augmented cell death (indicated by the proportion of cells with sub-G1 DNA content, Fig. 6A). Concurrent analysis of pHH3 did not demonstrate any significant differences between treatment conditions for pHH3-positive cells with 4N or < 4N DNA content, arguing against a role for unscheduled mitosis underlying the combination effects in these cells. Extending these analyses, cell cycle analysis of PDX#4 (*TP53wt*), PDX#19-r (*TP53wt*), and PDX#10-r (*TP53mut*) corroborated an enhancement of cell death over cell cycle arrest upon exposure to the adavosertib-cytarabine combination as compared to single agents, independent of *TP53* mutation status (Additional file 1: Fig. S7). Concurrent analyses of pHH3 at 48 h showed no changes in pHH3-positive cells with < 4N DNA content in any treatment condition (Additional file 1: Fig. S7).

We explored the molecular basis for the synergistic interaction of WEE1 inhibition with cytarabine in the NALM6 *TP53*^+*/*+^ cells. Combination treatment led to a similar decrease in CDK2 and CDK-Y15 phosphorylation as compared to adavosertib treatment alone (Fig. 6B), consistent with anticipated on-target activity of adavosertib. Consistent with the S-phase cell cycle arrest induced by cytarabine (Fig. 6A), inhibitory CDK-pY15 phosphorylation was enhanced by exposure to cytarabine (Fig. 6B). Combination treatment also led to a greater increase in cleaved PARP (Asp214) and a significant reduction in total WEE1 protein levels. We extended these observations by showing a similar pattern of molecular responses in three relapsed PDX samples (Fig. 6C). CDK-Y15 phosphorylation was substantially diminished in ALL blasts exposed to adavosertib either as a single agent or in combination, but was maintained or even enhanced upon exposure to cytarabine. At this 24 h time point, ALL blasts exposed to adavosertib alone and in combination with cytarabine also exhibited enhanced apoptosis as compared to vehicle or cytarabine alone, as evidenced by enhanced cleaved PARP protein levels.

Collectively, these data suggest that *TP53* mutations do not predispose ALL cells to undergo forced mitotic entry in response to adavosertib or its combination with cytarabine despite on-target WEE1 kinase inhibition, but combination treatment favors induction of apoptotic cell death over cell cycle arrest.

## Discussion

In this study, we demonstrate the anti-leukemic activity of the selective WEE1 kinase inhibitor adavosertib in a large majority of high-risk and relapsed ALL specimens, independent of *TP53* mutation status. We show that treatment with adavosertib results in S-phase disruption even in the absence of standard chemotherapeutic DNA-damaging agents and that premature mitotic entry is not required for its anti-leukemic activity; though abrogation of the G2-M checkpoint is still involved. We further demonstrate that WEE1 inhibition enhances the anti-leukemic effects of multiple conventional chemotherapeutics used in the relapsed or re-induction treatment setting for ALL. In particular, we highlight the heightened chemosensitivity to cytarabine by combined WEE1 inhibition in which ALL blasts preferentially promote cell death over cell cycle arrest. While WEE1 inhibition has been studied previously in ALL, to our knowledge, this is the first report to document the functional importance of WEE1 in primary cultures of relapsed ALL and define it as a potential p53 independent therapeutic target for the improved treatment of high-risk and relapsed ALL.

Previous reports using adavosertib in combination with DNA-damaging chemotherapeutics or radiotherapy demonstrated p53-deficient cancer cells to be more susceptible to WEE1 inhibition [17–19]; such that *TP53* mutation status has been cited as a biomarker of response in clinical studies of adavosertib [33]. However, in line with several other cancer types including sarcoma [31] and acute myeloid leukemia [20], our results indicate that *TP53* mutation status is not predictive of adavosertib response in high-risk and relapsed ALL. In fact, adavosertib exposure led to a significant decrease in leukemic blast survival in the vast majority of the primary and primary-derived ALL specimens investigated at concentrations safely attained in clinical studies [26, 27, 30], irrespective of *TP53* mutation status and in the absence of any additional exogenous stress. This observation is of substantial therapeutic relevance, ascribing a critical role of WEE1 kinase activity in the survival of many relapsed ALL blasts and greatly extending the therapeutic utility of WEE1 kinase-targeting compounds and potential combinations to a wider treatment context. Although the precise mechanism(s) underlying this discrepancy remains to be determined, these results also underscore the requirement for more rigorous characterization of resistance profiles and robust biomarkers of response for the clinical implementation of WEE1 inhibitors since a subset of our specimens did not respond to adavosertib as a single agent at clinically relevant concentrations and sensitivity was independent of basal WEE1 expression levels. One potential resistance factor warranting investigation could be the functionally redundant WEE1 family protein PKMYT1, for example; upregulation of which has been shown to be an acquired resistance factor to adavosertib in solid tumors in vitro [34].

WEE1 is critical for the maintenance of genomic integrity both during DNA replication and prior to mitosis as gatekeeper of the G_2_-M transition. Consistent with these roles, we observed substantial S-phase arrest in response to single agent adavosertib and abrogation of the G_2_-M checkpoint with enhanced mitotic entry; though, importantly, this was not associated with abnormal mitoses. Further implicating the crucial role of WEE1 during S-phase, we additionally identified promising combination activity of adavosertib with the S-phase acting nucleoside analog cytarabine. Prior studies by Tibes et al*.* [35] and other groups [16, 36] have identified cytarabine to be a candidate combination partner for adavosertib therapy in ALL but here we substantiate and extend these findings using numerous primary-derived relapsed and high-risk ALL specimens in a clinically-predictive co-culture model system [37]. Mechanistically, we demonstrate abrogation of cytarabine-induced S-phase arrest and heightened apoptosis in response to the combination, supporting a model by which concomitant on-target WEE1 inhibition further increases DNA damage and, as opposed to engaging cell cycle arrest, cells preferentially undergo apoptotic cell death. However, the role of WEE1 in G_2_-M phase should not be omitted mechanistically in potential drug combinations since substantial synergistic interaction was also observed with the anti-mitotic microtubule-targeting agent vincristine. Furthermore, there are developing preclinical and clinical interests in combination approaches incorporating other targeted compounds, such as CHK1 [38] and PARP1/2 inhibitors [39], or immunotherapies [40] which may ultimately help reduce toxicities associated with the use of chemotherapeutics.

Adavosertib is the most clinically advanced WEE1 inhibitor. As a monotherapy adavosertib is well tolerated with a favorable toxicity profile and, in combination with DNA-damaging chemotherapeutics, has demonstrated promising clinical activity in several studies of patients with treatment refractory solid tumors [19, 26]. For example, in a phase II study of adavosertib in combination with carboplatin for refractory *TP53* mutated ovarian cancer, overall response rates were more than doubled to 43% in this poor outcome cancer as compared to response rates of 11% to 21% for other second-line treatments [27]. The potency and safety profile of adavosertib have driven its rapid adoption into pediatric tumor clinical studies including patients with relapsed neuroblastoma, medulloblastoma, or rhabdomyosarcoma [33, 41]. In a phase 0 study for patients with glioblastoma, adavosertib was found to achieve pharmacologically relevant therapeutic concentrations in the central nervous system (CNS) [42]. Given that CNS disease recurrence is a major contributor to relapse in ALL [43], this further substantiates the therapeutic potential for adavosertib for high-risk and relapsed patients since sufficient drug penetration in the CNS is a prerequisite for effective treatment and could help counter CNS disease recurrence. As of this publication, no clinical studies of adavosertib have yet included patients with ALL. However, the continued development of additional WEE1 targeting therapeutics, including WEE1 inhibitor ZN-c3 [44] and the WEE1 degrader ZNL 02-0096 [45], along with trials of adavosertib with cytarabine in AML (NCT02666950) may help pave the way for WEE1-targeting clinical studies for ALL.

Future studies will be required to determine the in vivo effectiveness of this therapeutic approach whereby blasts are arguably more proliferative and could be even more susceptible to the anti-leukemic effects of WEE1 inhibition by adavosertib. Irrespective of this, our work greatly corroborates findings that WEE1 kinase activity is critical for maintaining genomic integrity in proliferating ALL blasts and substantiate WEE1 inhibition as a viable p53-independent therapeutic strategy for high-risk and relapsed ALL that should be considered for future clinical evaluation.

## Supplementary Information

**Additional file 1: Table S1.** Patient characteristics of patient-derived xenograft (PDX#) and primary ALL (ALL#) samples. **Table S2.** Antibodies used for flow cytometry and immunoblotting. **Table S3.** *TP53 *mutations identified by targeted Sanger sequencing. **Table S4.** Primer sequences and PCR reaction conditions for Sanger sequencing of *TP53 *exons 4-8. **Figure S1.** WEE1 inhibitor AZD1775 induces on-target apoptotic cell death and WEE1 kinase inhibition in a *TP53 *isogenic model. **Figure S2.** Adavosertib does not adversely affect proliferation of supporting mesenchymal stem cells. **Figure S3.** AZD1775 does not force mitotic catastrophe in ALL blasts as a single agent in vitro. **Figure S4.** Enhanced expression of replicative stress marker γH2AX in response to AZD1775 is diminished by pan-caspase inhibition in NALM6 ALL cells. **Figure S5.** AZD1775 sensitizes ALL blasts to chemotherapeutic drug classes used in the relapsed ALL clinical setting. **Figure S6.** AZD1775 sensitizes ALL cells to the relapse-specific nucleoside analog clofarabine. **Figure S7. **AZD1775 augments cell death induced by cytarabine in high-risk and relapsed ALL PDX samples in vitro. **Figure S8.** p53 is functionally inactive in hypodiploid sample PDX#11.

## Data Availability

The dataset supporting the conclusions of this article is available from the corresponding author upon request.

## Abbreviations

ALL: Acute lymphoblastic leukemia
ANOVA: Analysis of variance
AraC: Cytarabine (cytosine arabinoside)
ATP: Adenosine triphosphate
CDK1/2: Cyclin-dependent kinase (1/2)
CHK1: Checkpoint kinase 1
CNS: Central nervous system
DMSO: Dimethyl sulfoxide
EDTA: Ethylenediaminetetraacetic acid
FBS: Fetal bovine serum
FITC: Fluorescein isothiocyanate
HRP: Horseradish peroxidase
hTERT: Human telomerase reverse transcriptase
IC50: Concentration causing 50% growth inhibition
MDM2: Mouse double minute 2
NSG: NOD.Cg-Prkdc^scid^Il2rg^tm1Wjl^/SzJ
PARP: Poly-ADP ribose polymerase
PDX: Patient-derived xenograft
pHH3: Phosphorylated (Ser-10) histone H3
PI: Propidium iodide
PKMYT1: Protein kinase, membrane associated tyrosine/threonine 1
RIPA: Radioimunnoprecipitation assay
STR: Short-tandem repeat
zVAD.fmk: Carbobenzoxy-valyl-alanyl-aspartyl-[O-methyl]- fluoromethylketone
γH2AX: Phosphorylated (Ser-139) histone H2A
