# Correction: Targeting WEE1 kinase as a p53-independent therapeutic strategy in high-risk and relapsed acute lymphoblastic leukemia

**DOI:** 10.1186/s12935-025-04021-4

**Published:** 2025-11-07

**Authors:** Hayden L. Bell, Helen J. Blair, Mankaran Singh, Anthony V. Moorman, Olaf Heidenreich, Frederik W. van Delft, John Lunec, Julie A. E. Irving

**Affiliations:** 1 Wolfson Childhood Cancer Research Centre, Translation and Clinical Research Institute Newcastle University Centre for Cancer , Newcastle Upon Tyne, UK; 2 Princess Máxima Center for Pediatric Oncology, Utrecht, The Netherlands; 3 Bioscience Institute Newcastle University Centre for Cancer , Newcastle Upon Tyne , UK

**Correction: Cancer Cell International (2023) 23:202**

10.1186/s12935-023-03057-8

In this article [1], Fig. 6 appeared incorrectly and have now been corrected in the original publication. For completeness and transparency, the old incorrect and correct versions are displayed below. The original article has been corrected.

Incorrect Fig. 6:



**Fig**. **6** AZD1775 abrogates the intra-S-phase checkpoint and augments cell death induced by cytarabine. **A** A *TP53* isogenic NALM6 cell line model was treated for 24 h with AZD1775 (200 nM), AraC (15 nM), or their combination and subjected to dual cell cycle and pHH3 (mitotic cells) analysis using flow cytometry. Data are representative of three independent experiments. 2 N DNA content indicates cells in G0 or G1 phase. 4 N DNA content indicates cells in either G2 or M phase. Mitotic index was determined for cells with 4 N and < 4 N DNA content. Boxes indicate pHH3 + populations. Error bars show mean $\pm$ SD of at least three independent experiments. Top, PI alone; bottom, pHH3/PI. **B** Immunoblot of apoptotic and cell cycle markers in NALM6 cells treated for 24 h with AZD1775 (200 nM), AraC (15 nM), or their combination. Images are representative of three independent experiments. **C** Immunoblot of apoptotic and cell cycle markers in three relapsed PDX samples treated with respective IC50s of AZD1775, AraC, or their combination for 24 h. *N* = 1 for each respective sample. In **B** and **C**, ɑ-tubulin was used as loading control

Correct Fig. 6:

**Fig. 6:**
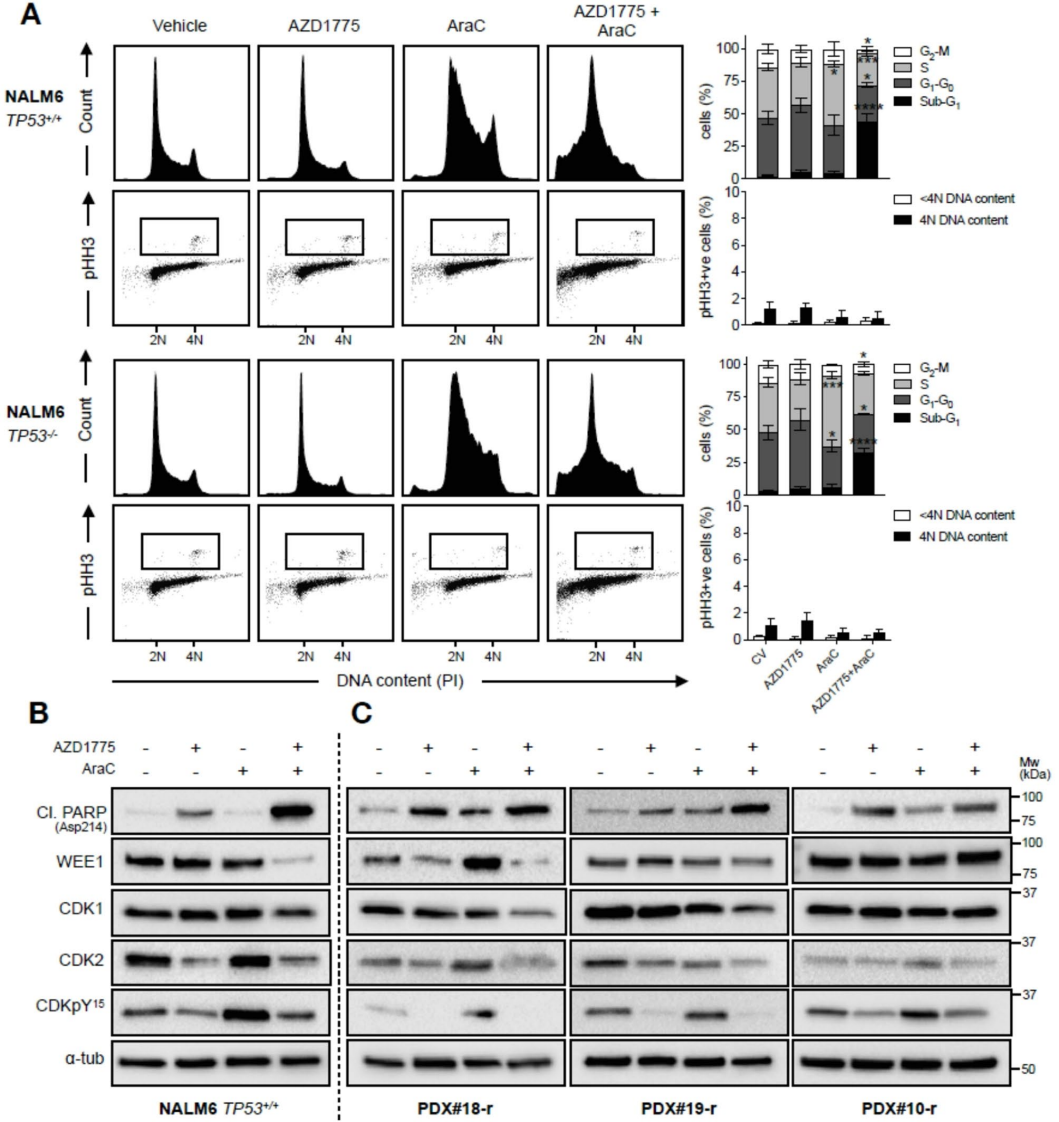
AZD1775 abrogates the intra-S-phase checkpoint and augments cell death induced by cytarabine. **A** A *TP53* isogenic NALM6 cell line model was treated for 24 h with AZD1775 (200 nM), AraC (15 nM), or their combination and subjected to dual cell cycle and pHH3 (mitotic cells) analysis using flow cytometry. Data are representative of three independent experiments. 2 N DNA content indicates cells in G0 or G1 phase. 4 N DNA content indicates cells in either G2 or M phase. Mitotic index was determined for cells with 4 N and < 4 N DNA content. Boxes indicate pHH3 + populations. Error bars show mean $\pm$ SD of at least three independent experiments. Top, PI alone; bottom, pHH3/PI. **B** Immunoblot of apoptotic and cell cycle markers in NALM6 cells treated for 24 h with AZD1775 (200 nM), AraC (15 nM), or their combination. Images are representative of three independent experiments. **C** Immunoblot of apoptotic and cell cycle markers in three relapsed PDX samples treated with respective IC50s of AZD1775, AraC, or their combination for 24 h. *N* = 1 for each respective sample. In **B** and **C**, ɑ-tubulin was used as loading control.
