# Supplementary material for: Targeting WEE1 kinase as a p53-independent therapeutic strategy in high-risk and relapsed acute lymphoblastic leukemia
Source: Cancer Cell Int. 2023 Sep 15;23:202. doi: 10.1186/s12935-023-03057-8 (PMC10502974; doi:10.1186/s12935-023-03057-8)
Supplement: Supplementary file 1 — Additional file 1: Table S1. Patient characteristics of patient-derived xenograft (PDX#) and primary ALL (ALL#) samples. Table S2. Antibodies used for flow cytometry and immunoblotting. Table S3. TP53 mutations identified by targeted Sanger sequencing. Table S4. Primer sequences and PCR reaction conditions for Sanger sequencing of TP53 exons 4-8. Figure S1. WEE1 inhibitor AZD1775 induces on-target apoptotic cell death and WEE1 kinase inhibition in a TP53 isogenic model. Figure S2. Adavosertib does not adversely affect proliferation of supporting mesenchymal stem cells. Figure S3. AZD1775 does not force mitotic catastrophe in ALL blasts as a single agent in vitro. Figure S4. Enhanced expression of replicative stress marker γH2AX in response to AZD1775 is diminished by pan-caspase inhibition in NALM6 ALL cells. Figure S5. AZD1775 sensitizes ALL blasts to chemotherapeutic drug classes used in the relapsed ALL clinical setting. Figure S6. AZD1775 sensitizes ALL cells to the relapse-specific nucleoside analog clofarabine. Figure S7. AZD1775 augments cell death induced by cytarabine in high-risk and relapsed ALL PDX samples in vitro. Figure S8. p53 is functionally inactive in hypodiploid sample PDX#11. [file 12935_2023_3057_MOESM1_ESM.pdf]

Additional file 1 for "Targeting WEE1 kinase as a p53-independent therapeutic strategy in high-risk and relapsed acute lymphoblastic leukemia"

Hayden L. Bell, et al.

1. ADDITIONAL METHODS
2. ADDITIONAL TABLES AND LEGENDS
3. ADDITIONAL FIGURES AND LEGENDS
4. ADDITIONAL REFERENCES

1. ADDITIONAL METHODS

Functional assessment of p53 activity

Drug sensitivity to p53-MDM2 antagonist idasanutlin (RG7388) was determined for primary and PDX ALL blasts as described in methods. Samples with idasanutlin IC50 values <1μM were considered sensitive (1), and samples with IC50 values >1μM were subjected to targeted Sanger sequencing of *TP53* exons 4 to 8 as described below. For further functional assessment of *TP53* aberration status, ALL blasts were treated with vehicle or idasanutlin (0.5 μM, 5μM) for 6 hours before p53 signaling pathway protein analysis by immunoblotting.

Targeted *TP53* mutation sequencing

Genomic DNA was extracted from mononuclear preparations of primary or patient-derived xenograft samples using a QIAamp DNA Mini Kit (#51304, Qiagen, Manchester, UK) and primers for *TP53* exons 4 to 8 were used to amplify the DNA by PCR using an AmpliTaq Gold DNA Polymerase DNA kit (#4311806, ThermoFisher Scientific). Primer sequences and thermal cycling conditions for individual amplicons are shown in Additional file 1: Table S4. Following agarose gel electrophoresis to verify amplicon size and PCR clean-up (QIAquick PCR purification kit, #28104, Qiagen), amplicons were outsourced to Source Bioscience (Cambridge, UK) for Sanger sequencing using both forward and reverse primers. The analysis of chromatograms and alignment with human *TP53* NCBI reference sequence (NM_000546.6) was conducted with Snap Gene v6.2 Software (Boston, MA; RRID:SCR_015052).

2. ADDITIONAL TABLES AND LEGENDS

Table S1. Patient characteristics of patient-derived xenograft (PDX#) and primary ALL (ALL#) samples.

Sample ID	Lineage	Age at Presentation (years)	Sex[a]	Disease status	Subtype	*TP53* mutation status[b]	Adavosertib IC50 (nM)
PDX#1	B-ALL	18	M	Presentation	*KMT2A*-rearranged	wt	180
PDX#2	B-ALL	50	F	Presentation	*KMT2A*-rearranged	wt	217
PDX#3	B-ALL	18	M	Relapse	*BCR::ABL1*	wt	1140
PDX#4	B-ALL	16	F	Presentation	*TCF3::HLF*	wt	271
PDX#5	B-ALL	14	M	1st Relapse, 2nd Relapse	*TCF3::HLF*	wt	>10000, 1569
PDX#6	B-ALL	5	M	Presentation	High hyperdiploidy	wt	317
PDX#7	B-ALL	3	F	Relapse	High hyperdiploidy	wt	886
PDX#8	B-ALL	6	M	Relapse	High hyperdiploidy	wt	287
PDX#9	B-ALL	7	M	Relapse	High hyperdiploidy	wt	483
PDX#10	B-ALL	11	M	Relapse	Low hypodiploidy	mut	136
PDX#11	B-ALL	72	M	Relapse	Low hypodiploidy	mut	2190
PDX#12	B-ALL	6	F	Relapse	*ETV6::RUNX1*	wt	>10000
PDX#13	B-ALL	2	M	Presentation	*ETV6::RUNX1*	wt	1389
PDX#14	B-ALL	3	F	Relapse	*ETV6::RUNX1*	wt	>10000
PDX#15	B-ALL	1	F	Relapse	*ETV6::RUNX1*	wt	228
PDX#16	B-ALL	8	F	Relapse	iAMP21	wt	>10000
PDX#17	B-ALL	16	F	Presentation	*ZYMND8::PDGFRB*	wt	720
PDX#18	B-ALL	15	F	Relapse	Other - 46, XX, t(1;9) - (q2?5;q34) [7]	wt	866
PDX#19	B-ALL	64	F	Relapse	Other - 46,XX[15]	wt	324
PDX#20	T-ALL	18	M	Presentation	Failed - 46,XY[20]	wt	180
PDX#21	T-ALL	15	M	Presentation	*FIP1L1::PDGFRA*	wt	1550
PDX#22	T-ALL	7	M	Presentation	*CEP128::PDGFRB*	wt	4445
ALL#1	B-ALL	7	F	Presentation	High hyperdiploidy	wt	395
ALL#2	B-ALL	10	M	Relapse	*IGH*-rearranged	mut	>10000
ALL#3	B-ALL	1	M	Presentation	Other	wt	616
ALL#4	T-ALL	13	M	Presentation	*PDGFRB* deletion	wt	438

[a] M, male; F, female.

[b] wt, wildtype; mut, mutant. See Additional File 1: Table S3 for *TP53* mutations identified by targeted Sanger sequencing.

Table S2. Antibodies used for flow cytometry and immunoblotting.

Antibody[a]	Clone	Catalog no.	RRID	Manufacturer
		Antibodies for flow cytometry		
anti-mCD45 PE-Cy7	30-F11	552848	AB_394489	BD Biosciences
anti-hCD45 APC-H7	2D1	560178	AB_1645479	BD Biosciences
anti-hCD10 PE	HI10a	555375	AB_395776	BD Biosciences
anti-hCD19 APC	SJ25C1	561742	AB_10894000	BD Biosciences
anti-hCD34 PerCP	8G12	340430	AB_400034	BD Biosciences
anti-CD7 APC	CD7-6B7	561604	AB_10893354	BD Biosciences
anti-CD5 PerCP Cy5.5	L17F12	341109	AB_2868765	BD Biosciences
anti-p-histone H3 (Ser10) Alexa Fluor 488	D2C8	3465	AB_10695860	CST
		Antibodies for immunoblotting		
α-tubulin	B-5-1-2	T6074	AB_477582	Sigma-Aldrich
cyclin B1	GNS1	sc-245	AB_627338	Santa Cruz
CDK1 (cdc2)	POH1	9116	AB_2074795	CST
CDK2	78B2	2546	AB_2276129	CST
Cleaved PARP (Asp214)	-	9541	AB_331426	CST
MDM2	IF2	OP46	AB_437744	Merck Millipore
p21	SX118	556430	AB_396414	BD Biosciences
p53	DO-1	sc-126	AB_628082	Santa Cruz
p-CDK1-Y15 (cdc2-pY15)	-	9111	AB_331460	CST
p-Histone H2A.X (Ser139)	20E3	9718	AB_2118009	CST
WEE1	D10D2	13084	AB_2713924	CST

[a] m, mouse; h, human; CST, Cell Signaling Technology.

Table S3. ***TP53*** **mutations identified by targeted Sanger sequencing.**

Sample ID	Mutation
ALL#2-relapse	TP53:NM_000546.6:exon7:c.T721C:p.S241P
PDX#10-relapse	TP53:NM_000546.6:exon6:c.A659G:p.Y220C
PDX#11[a]	TP53:NM_000546.6:exon4:c.C357G:p.A119A

[a] See Additional file 1: Fig. S8 for functional assessment of p53 activity.

Table S4. Primer sequences and PCR reaction conditions for Sanger sequencing of *TP53* exons 4-8.

Target Region	Primer Sequences[a]	PCR reaction conditions[b]
TP53 exon 4	F 5'-CCTGGTCCTCTGACTGCTCT-3'<br>R 5'-GCCAGGCATTGAAGTCTCAT-3'	14 touchdown cycles + 20 standard cycles:<br>D 94°C 20s<br>A 57°C 60s<br>E 72°C 60s
TP53 exon 5	F 5'-GGATCCATCTGTTCACTTGTGCCCTG-3'<br>R 5'-GAATTCAACCAGCCCTGTCGTCTCTC-3'	14 touchdown cycles + 20 standard cycles:<br>D 94°C 20s<br>A 55°C 60s<br>E 72°C 60s
TP53 exon 6	F 5'-GCCTCTGATTCCTCACTGAT-3'<br>R 5'-GGAGGGCCACTGACAACCA-3'	14 touchdown cycles + 20 standard cycles:<br>D 94°C 20s<br>A 55°C 60s<br>E 72°C 60s
TP53 exon 7	F 5'-GGATCCAGGCGCACTGGCCTCATCTT-3'<br>R 5'-GAATTCAGGGGTCAGAGGCAAGCAGA-3'	14 touchdown cycles + 20 standard cycles:<br>D 94°C 20s<br>A 60°C 60s<br>E 72°C 60s
TP53 exon 8	F 5'-GAGCCTGGTTTTTTAAATGG-3'<br>R 5'-TTTGGCTGGGGAGAGGAGCT-3'	14 touchdown cycles + 20 standard cycles:<br>D 94°C 20s<br>A 60°C 60s<br>E 72°C 60s

[a] F, forward; R, reverse.

[b] initial denaturation was performed at 95°C for 10 minutes and the last cycle was followed by a final extension step at 72°C for 5 minutes in all cases. Touchdown cycles started at 7°C above annealing temperature and decreased by 0.5°C per cycle. D, denaturation; A, annealing; E, extension.

3. ADDITIONAL FIGURES AND LEGENDS

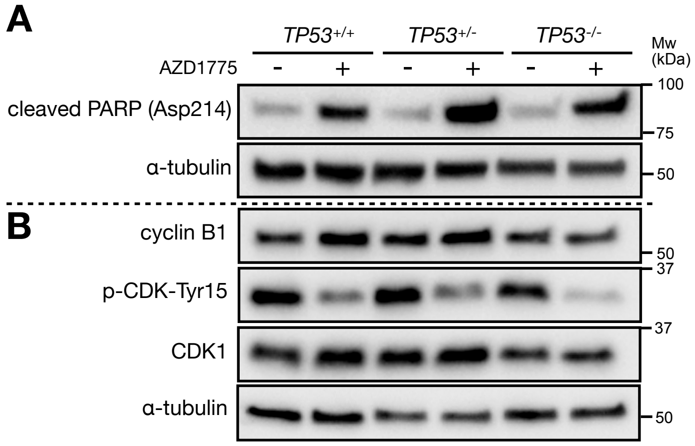

Figure S1. WEE1 inhibitor AZD1775 induces on-target apoptotic cell death and WEE1 kinase inhibition in a *TP53* isogenic model. (**A**) Immunoblot of apoptotic marker cleaved PARP (Asp213) in a NALM6 *TP53* isogenic model treated with DMSO or AZD1775 (200nM) for 24 hours. (**B**) Immunoblot of cell cycle markers in a NALM6 *TP53* isogenic model treated with DMSO or AZD1775 (200nM) for 6 hours. In **A** and **B**, α-tubulin was used as loading control and images are representative of three independent experiments.

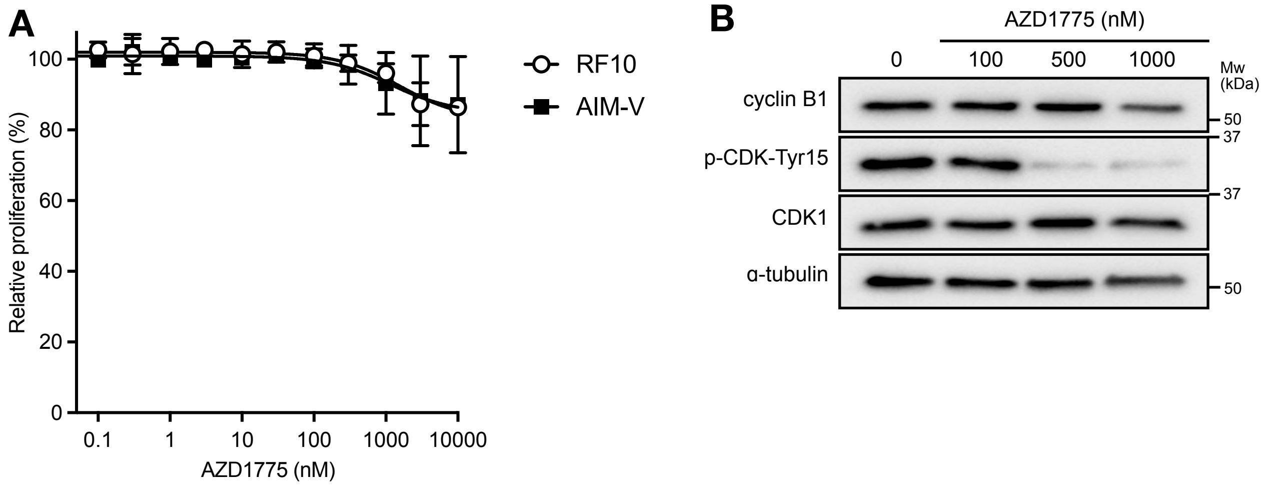

Figure S2. Adavosertib does not adversely affect proliferation of supporting mesenchymal stem cells (**A**) Dose-response curves of hTERT-immortalized MSCs cultured in RPMI with 10% FBS (RF10) or serum-free AIM-V cell culture media, optimized for survival of MSCs or leukemic blasts respectively, exposed to AZD1775 for 96 h. Data were normalized to DMSO and represent mean±SEM of three independent experiments. **b** Immunoblots showing reduced p-CDK-Tyr15 following 6 h exposure to increasing concentrations of AZD1775. α-tubulin was used as loading control and images are representative of three independent experiments.

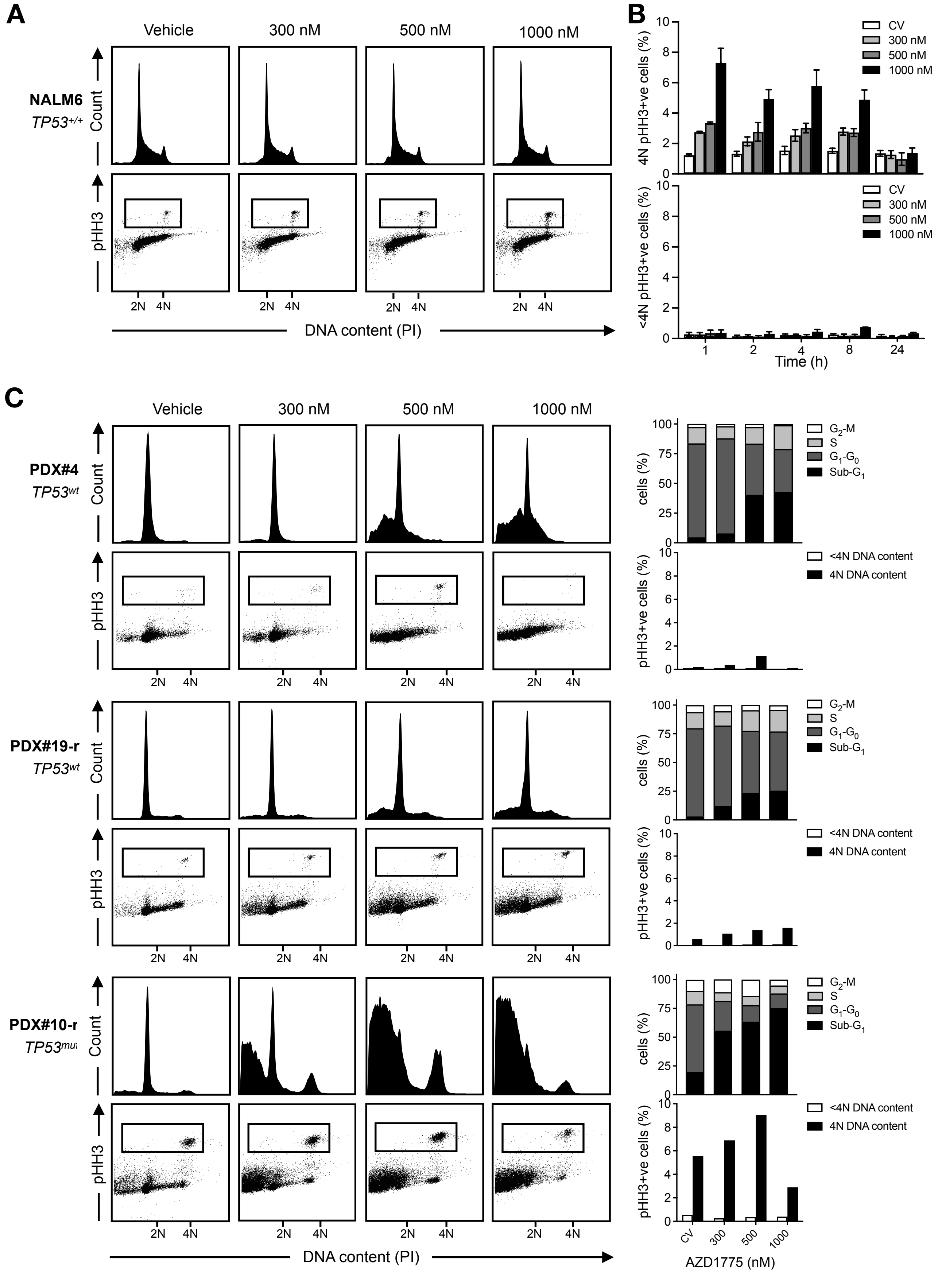

Figure S3. AZD1775 does not force mitotic catastrophe in ALL blasts as a single agent *in vitro*. (**A**) Representative dual cell cycle and pHH3 analysis of NALM6 cells treated with DMSO or increasing doses of AZD1775 for 1 hour. 2N DNA content indicates cells in G_1 or G_1 phase. 4N

(Figure S3 legend, continued)

DNA content indicates cells in either G_2 or M phase. Data are representative of three independent experiments. (**B**) Time course analysis to determine mitotic index with 4N and <4N DNA content was performed in NALM6 cells for 1 to 24 hours following treatment with DMSO or increasing concentrations of AZD1775. Error bars show mean±SEM of three independent experiments. (**C**) Dual cell cycle and pHH3 analysis was performed in high-risk *TCF3-HLF* rearranged PDX#4 (*$TP53^{wt}$*), relapsed B-other PDX#19-r (*$TP53^{wt}$*) and relapsed hypodiploid PDX#10-r (*$TP53^{mut}$*) samples treated with increasing DMSO or increasing doses of AZD1775 for 48 hours. Data are representative of one independent experiment for each sample. In all panels, mitotic index was determined for cells with 4N and <4N DNA content and boxes indicate $pHH3^+$ populations. Top, PI alone; bottom, pHH3/PI.

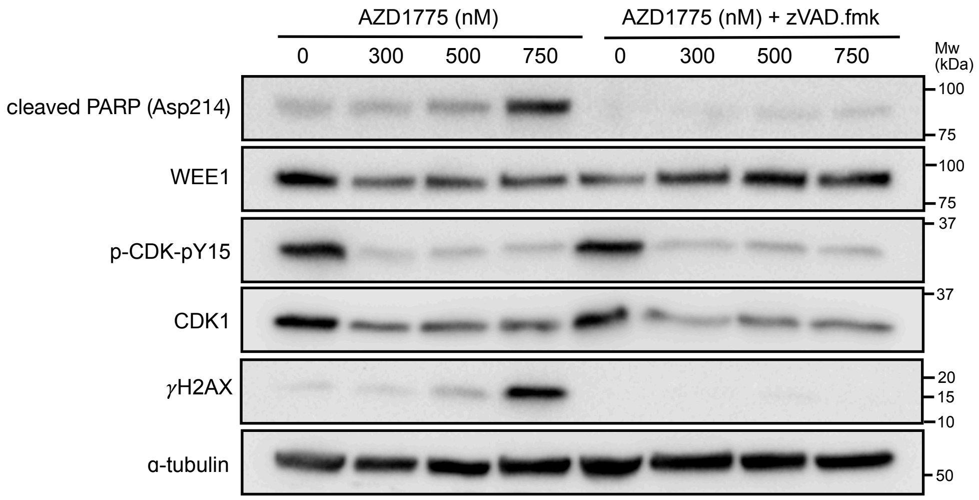

Figure S4. Enhanced expression of replicative stress marker γH2AX in response to AZD1775 is diminished by pan-caspase inhibition in NALM6 ALL cells. Immunoblot of apoptotic marker cleaved PARP (Asp214), replication stress marker γH2AX, and cell cycle markers in NALM6 cells treated with DMSO or increasing concentrations of AZD1775 for 24 hours, with or without pretreatment with pan-caspase inhibitor zVAD.fmk (25 μM) for 6 hours. α-tubulin was used as a loading control and images are representative of three independent experiments.

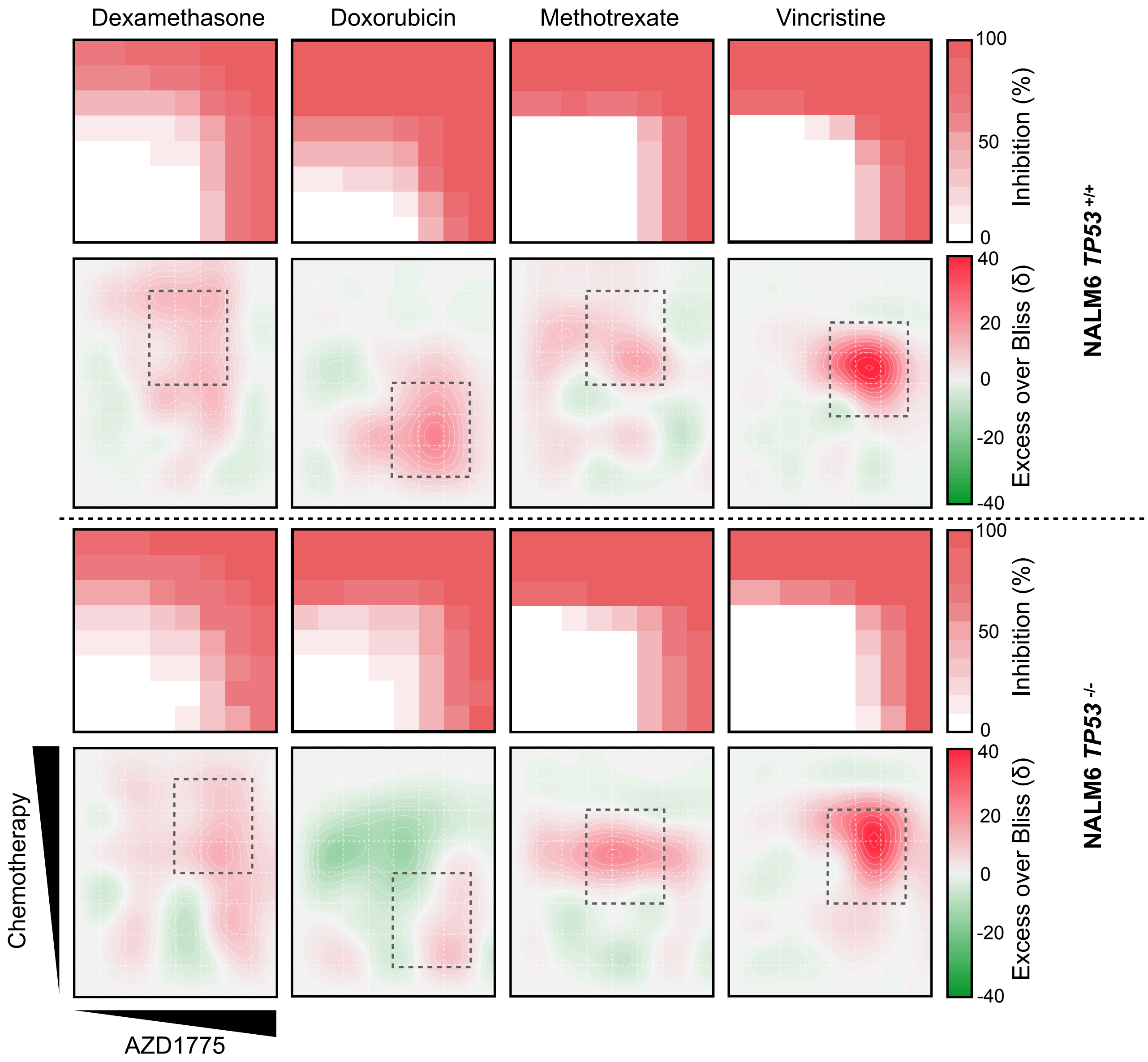

Figure S5. AZD1775 sensitizes ALL blasts to chemotherapeutic drug classes used in the relapsed ALL clinical setting. Representative dose-response matrix analyses showing cell inhibition and synergistic landscape across diverse pairwise AZD1775-chemotherapy (dexamethasone, doxorubicin, methotrexate, or vincristine) dose combinations in a NALM6 *TP53* isogenic model. Grey dashed boxes indicate the most synergistic area. Data are representative of three independent experiments in technical triplicate. Data for the AZD1775-AraC combination are in **Fig. 4A**. Most synergistic area scores are reported in **Fig. 4B**.

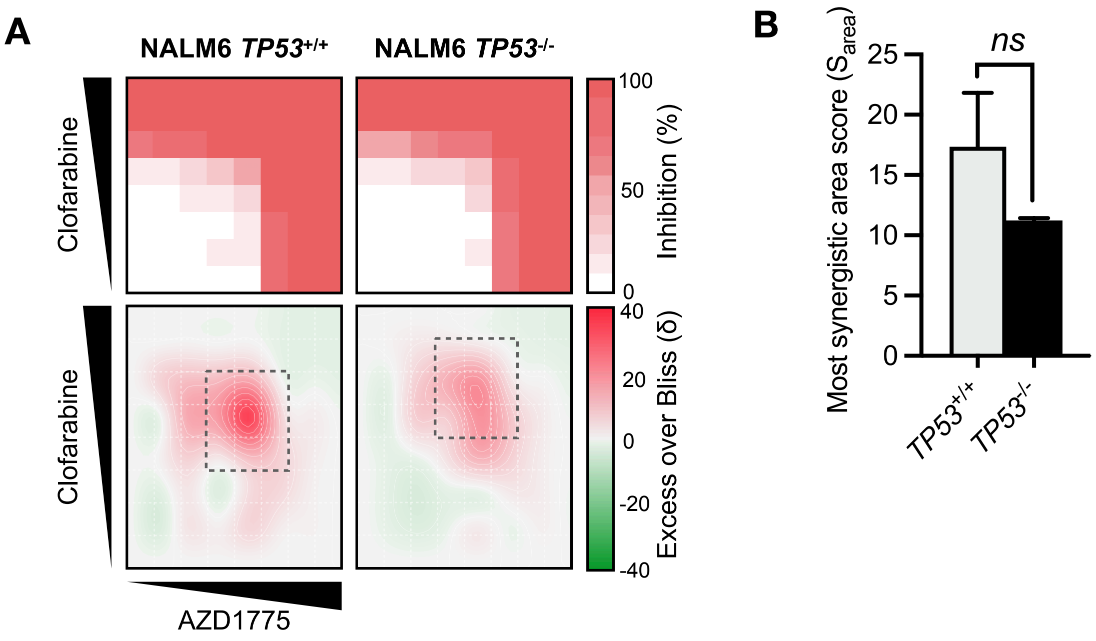

Figure S6. AZD1775 sensitizes ALL cells to the relapse-specific nucleoside analog clofarabine. (**A**) Representative dose-response matrix analyses showing cell inhibition (top) and synergistic landscape (bottom) across diverse AZD1775-clofarabine dose combinations after 96 hours in a NALM6 *TP53* isogenic model. Grey dashed boxes indicate the most synergistic area. (**B**) Synergistic effects of the AZD1775-clofarabine combination were quantified in NALM6 *TP53*$^{+/+}$ and TP53$^{-/-}$ isogenic cells. Error bars indicate mean±SD of three independent experiments in technical technical triplicate.

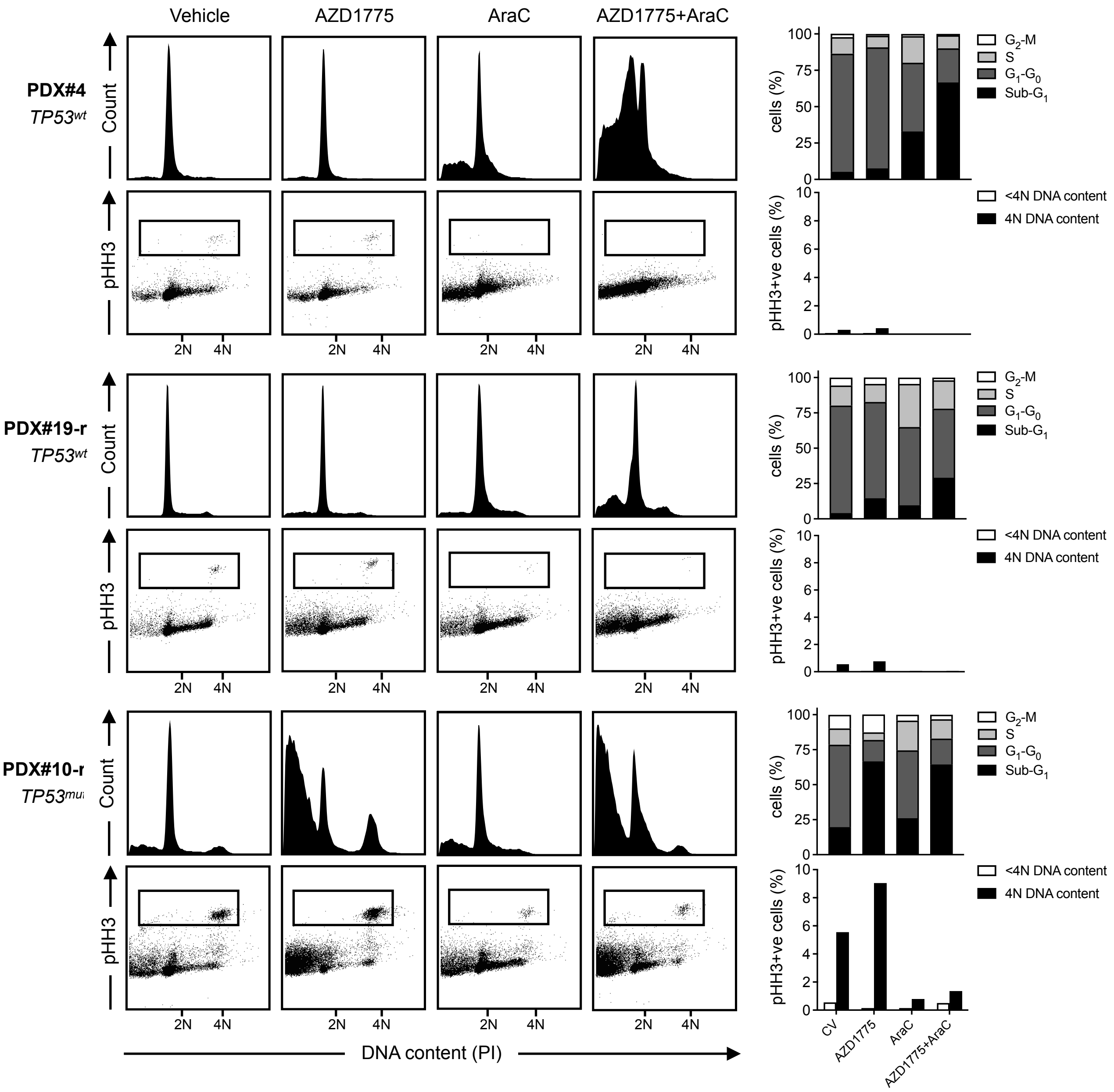

Figure S7. AZD1775 augments cell death induced by cytarabine in high-risk and relapsed ALL PDX samples *in vitro*. Dual cell cycle and pHH3 analysis was performed in high-risk *TCF3-HLF* rearranged PDX#4 (*TP53^{wt}*), relapsed B-other PDX#19-r (*TP53^{wt}*) and relapsed hypodiploid PDX#10-r (*TP53^{mut}*) samples treated with respective IC50s of AZD1775, AraC, or their combination for 48 hours. Hypodiploid cell DNA content (<2N) was normalized to diploid DNA content (2N). Top, PI alone; bottom, pHH3/PI. 2N DNA content indicates cells in G_1 or G_1 phase and 4N DNA content indicates cells in either G_2 or M phase. Mitotic index was determined for cells with 4N and <4N DNA content. Boxes indicate pHH3$^+$ populations. *N*=1 independent experiment.

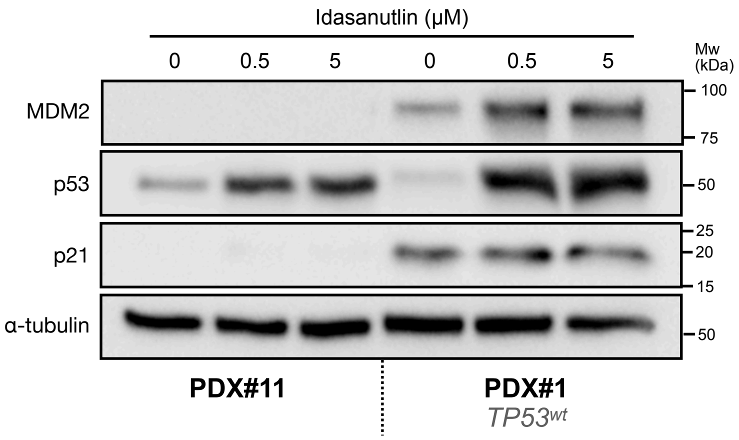

Figure S8. p53 is functionally inactive in hypodiploid sample PDX#11. Immunoblot of p53 and transcriptionally-regulated p53 target gene products MDM2 and p21 in PDX#11 and PDX#1 (*TP53^{wt}* control) cells treated with DMSO or increasing concentrations of p53-MDM2 antagonist idasanutlin for 6 hours. α-tubulin was used as a loading control and images are representative of one independent experiment.

4. ADDITIONAL REFERENCES

1. Bell H, Singh M, Blair H, van Delft F, Moorman A, Lunec J, et al. Preclinical Investigation of the p53-MDM2 Antagonist Idasanutlin (RG7388) Demonstrates Significant Activity in High Risk Adult Acute Lymphoblastic Leukemia. Blood. 2020;136:38.
